# Demand characteristics challenge effects in embodiment and presence

**DOI:** 10.1038/s41598-022-18160-5

**Published:** 2022-08-18

**Authors:** Pierre-Pascal Forster, Harun Karimpur, Katja Fiehler

**Affiliations:** 1 Experimental Psychology, Justus Liebig University Giessen, Giessen, Germany; 2 Center for Mind, Brain and Behavior (CMBB), University of Marburg and Justus Liebig University, Giessen, Germany

**Keywords:** Human behaviour, Psychology

## Abstract

The sensations to own and control a body as well as being located in a body describe the relation between ourselves and our body, termed embodiment. Embodiment plays a central role in our everyday actions. However, its assessment is challenging. Recent findings suggest that measures on embodiment are confounded by demand characteristics and suggestibility. To investigate the impact of demand characteristics on embodiment and presence, we compared results from an online experiment measuring participants’ expectations, to the same experiment in virtual reality (VR). In the online experiment, participants watched a video of a participant performing the VR experiment. In the VR experiment, participants performed a soap-bubble-kicking task, which allowed the feelings of embodiment and presence to arise. We manipulated temporo-spatial movement synchrony (Movement: synchronous, asynchronous) and avatar visibility (Visibility: visible, invisible). In addition, we assessed participants’ suggestibility with exercises. The introduced manipulations influenced the ratings in both experiments similarly. Embodiment ratings were additionally affected by suggestibility. Altogether, our results show that participants were aware of the research hypotheses, which indicates that demand characteristics confound embodiment and presence research alike. Overcoming challenges of demand characteristics is crucial to correctly interpret scientific results and to translate these results into applied settings.

## Introduction

Regardless of the motor task we perform, from fine-tuned motor skills when playing an instrument to simple actions like pressing a key, we experience a direct relation to our body. This relation can be described as the feeling to own a body, to have a certain location relative to this body, and to be in control of its actions, defined as embodiment^1^. The rubber hand illusion (RHI) is frequently used to investigate embodiment. In this illusion [c.f.,^2^], participants see brush strokes on a rubber hand in front of their body, and perceive synchronous tactile feedback on their real hand, hidden from view. It is generally believed that embodiment emerges from the integration of multisensory information, in particular, visual information of the rubber hand and somatosensory information of the own hand^2,3^. In addition, specific constraints, like shape^4,5^ and orientation^4,6^, of the embodied rubber hand must be satisfied for embodiment to emerge. At the same time, body representations are also highly flexible. For example, virtually switching the left and right hand while interacting with a ball did not prevent the feeling of ownership to arise^7^. However, previous investigations on embodiment have recently been criticised to be influenced by factors not inherent to embodiment, namely demand characteristics and suggestibility^8,9^.

Clearly, hypotheses should not be known to participants, as their knowledge can influence their behaviour^10^. Participants’ expectations about what the experiment and the experimenter requires from them is known as demand characteristics^8,11,12^. Lush^8^ argues that demand characteristics can at least partly explain results in RHI experiments. In his study, participants examined materials explaining the experimental setup and rated their expected sensations on an RHI questionnaire. Crucially, they did not participate in the actual experiment, but answered the questions on the sole basis of their knowledge about the experiment. The results were indeed similar to standard RHI experiments, suggesting that demand characteristics might influence the subjective ratings on the RHI.

Since then, several studies have suggested that demand characteristics play indeed an important role in embodiment research^13,14^. Even frequently applied objective measures like proprioceptive drift, i.e. the perceived drift of the real hand position towards the rubber hand, and skin conductance are argued to be subject to demand characteristics^13^. The critique of demand characteristics extends beyond mere RHI experiments to other paradigms and constructs. For example, measuring participant’s simulator sickness before starting a virtual reality (VR) experiment increased simulator sickness scores when measured after the experiment^15^. However, demand characteristics do not always affect the results. Manipulating participants beliefs about the experimental hypothesis by providing differently framed instructions in an experiment on the facial feedback hypothesis did not abolish the effect^16^. It is therefore necessary to assess the extent to which demand characteristics influence subjective and objective measures, e.g. of embodiment.

On the one hand, demand characteristics could lead to a response bias, i.e. participants are consciously adjusting their responses to match the alleged expectations of the experimenter^10^. On the other hand, suggestible participants might be sensitive to unknowingly create experiences matching those of the perceived demands, which has been more recently described as phenomenological control^9,17^. In other words, knowing that ownership is expected to occur over a synchronously stroked rubber hand might lead to the sensation of ownership, because participants expect it to occur and unknowingly adjust their experiences accordingly. The concept of suggestibility is thus closely linked to demand characteristics, but different from imagination where participants are aware that they created these experiences by themselves^12^. According to this argumentation, participant’s experiences could fall in line with the perceived demands, possibly, but not necessarily, matching the research hypotheses^12^.

Previous work has shown that suggestibility can indeed predict results in embodiment experiments^9,18,19^. Whether and to what extent embodiment might depend on suggestibility is a matter of current debate^20,21^. Data pointing to suggestibility predicting embodiment^9^ was recently questioned by a reanalysis^20^. In this reanalysis, the sample was divided into quartiles depending on their suggestibility scores. The illusion was produced in all four subgroups, i.e. also for participants scoring very low on suggestibility. This result suggests that embodiment cannot be explained solely on the basis of suggestibility. However, excluding suggestible participants from the sample makes it less likely to find positive RHI results^19^. Altogether, suggestibility might have an influence on results of embodiment although results in embodiment seem not to be reducible to suggestibility effects.

In this study, we investigated the influence of demand characteristics and suggestibility on embodiment. In addition, we wanted to test whether the same critique of demand characteristics can be applied to presence, which we conceptualised as the sense of “being there” in an environment [c.f.,^22^]. Similar to embodiment research, it might be easy for participants to predict the experimental purpose of questionnaires together with experimental manipulations used in presence research. Therefore, it is likely that demand characteristics could also affect the sense of presence. We constructed an experiment in which we combined embodiment and presence measures. To examine the influence of demand characteristics, we performed the same experiment twice^11^: once in an online version testing participants’ expectations based on descriptions about the experiment without actually participating in the experiment, and once as a VR experiment where a second group of participants actively performed the task. In the online experiment, we presented the experimental manipulations to participants and let them rate their expected sensations of embodiment and presence. We constructed the VR experiment to have the exact same manipulations as depicted in the online experiment.

So far, presence has been mostly investigated in VR [e.g.,^23,24^]. Using VR enabled us to combine embodiment and presence in one single study. Comparing the results of the two experiments can further inform us if participants who only observed participants performing the VR experiment build expectations matching the responses of participants who actively performed the VR experiment. This would suggest that participants knew the research hypotheses and that their results could be affected by demand characteristics.

## Methods

This study consisted of two independent within-subject experiments, an online and a VR experiment, with two independent groups of participants (combined analysis using a mixed design). To avoid carry over effects from the online to the VR experiment or vice versa we conducted the experiments in two independent samples. The online experiment was run as an online version of the VR experiment by showing videos of a participant performing the VR experiment in the laboratory. Based on the videos, participants rated their expected experience on selected questionnaire items [c.f.,^8,11^]. In the following, we will describe the VR experiment first, and then explain the specifics of the online experiment.

### Experiment 1: Virtual reality

#### Participants

We performed a power analysis (GPower 3.1.9.6,^25^) on pilot data (n = 13) to estimate the required sample size based on the effect of Movement on the ownership (*d*_*z*_ = 1.01), agency (*d*_*z*_ = 1.24), location (*d*_*z*_ = 1.16) and presence (*d*_*z*_ = 0.47) ratings. The power was set to 0.8 and the α error to 0.05. This resulted in a required sample of 9 participants for the effect of ownership, 9 for location, 8 for agency and 40 for presence. Our final sample consisted of 44 participants (26 females and 18 males), with a mean age of 24 years (SD = 3, ranging from 19 to 33). We sampled more participants than required based on the power analysis to ensure that at least 40 participants remained in the final sample after applying the exclusion criteria. Participants were recruited via university e-mail and received 8€ per hour or course credits. To participate in the experiment, participants had to be 18–35 years old, speak German fluently, have intact 3D vision and no poor eyesight (correction with soft contact lenses up to two dioptre were allowed). In addition, participants with pathological impairments of the sensory or motor systems or known neuropsychological disorders were not allowed to participate in the experiment. For one participant the avatar did not match in skin colour and for another participant the experimenter wrongly chose the sex of the avatar. As previous studies showed that embodying such unmatched avatars is possible^26,27^, we decided to leave those participants in the sample. The experimenters followed the university’s hygiene rules due to the COVID-19 pandemic. The Giessen University Ethics Committee approved the study and all participants provided written informed consent before beginning the experiment. The experiment was conducted in accordance with the Declaration of Helsinki (2004).

#### Setup

**Figure 1:**
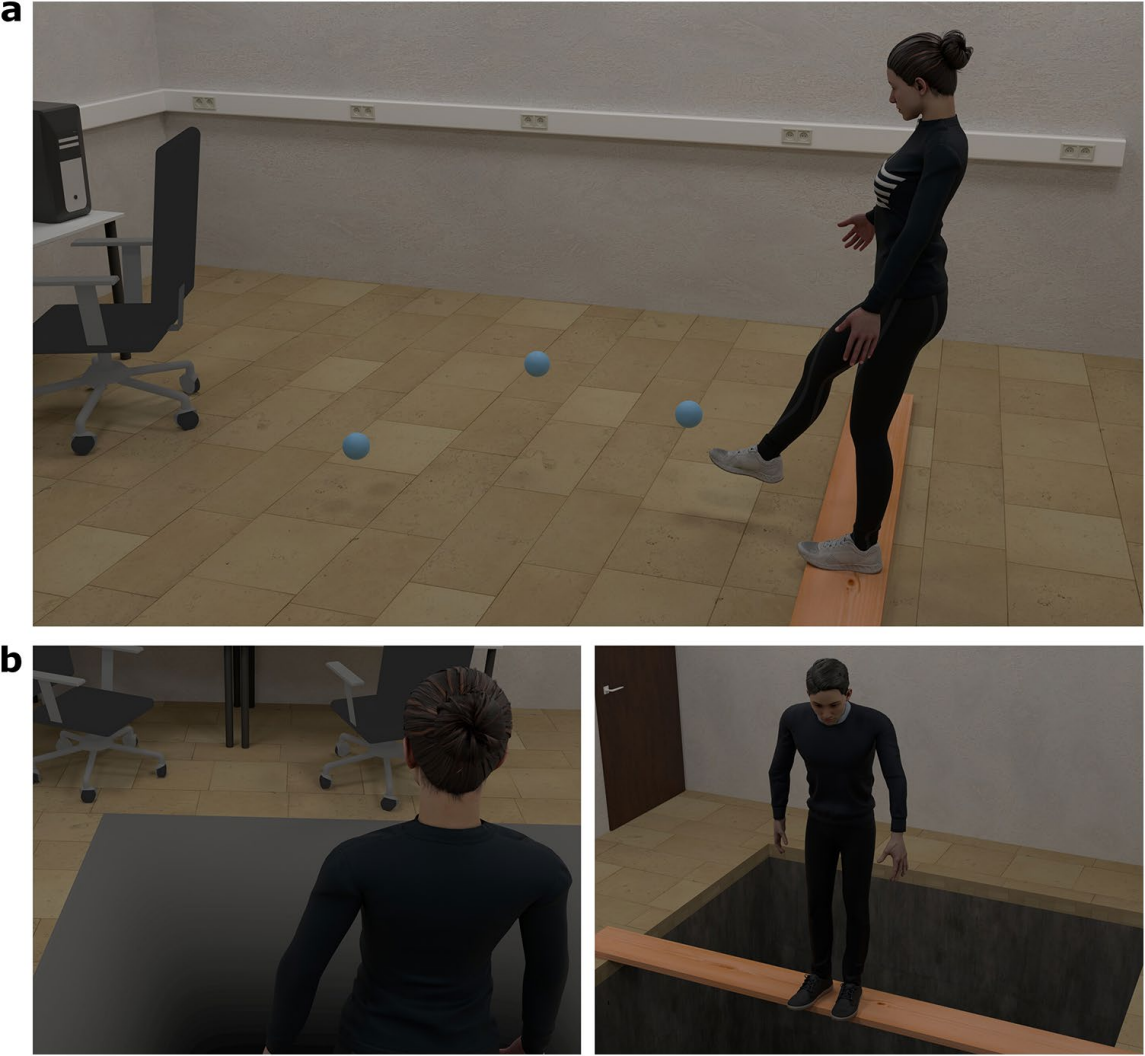
Virtual soap-bubble-kicking task and virtual presentation of the control and threat stimulus. (**a**) shows the soap-bubble-kicking task participants performed in VR. (**b**) shows the control (left) and threat (right) stimuli. Images are rendered from a third person perspective, but participants were always presented with the virtual environment from a first-person perspective. The avatar models are royalty free and taken from www.mixamo.com.

A Vive Pro Eye (HTC Corporation, Taoyuan City, Taiwan) head mounted display (HMD, 110° field of view, 1440 × 1600 pixels per eye, 90 Hz frame rate), trackers and a controller were used for the experiment. Skin conductance was continuously recorded at 2000 Hz with a BIOPAC MP36R system (BIOPAC Systems, Inc., Goleta, CA, USA). The virtual environment (Fig. 1) was modelled in Blender 2.93 and the experiment run in Vizard 6 (WorldViz, Santa Barbara, CA, USA). We modelled the virtual laboratory similar to the real laboratory. In the real laboratory, participants stood on the edge of a wooden plank. The plank was also presented in the virtual laboratory, both spatially coinciding. A gender matched avatar (Mixamo, as part of Adobe, San Jose, CA, USA) was used to represent the participant’s body in the virtual environment. Six Vive trackers captured the motion of participants’ arms, feet and torso. Participants held a Vive controller in their left hand to rate the questionnaire items.

#### Task and study design

Participants performed a soap-bubble-kicking task. In this task, they had to catch soap bubbles with their feet which burst after making contact (Fig. 1). As the soap bubbles always appeared in the lower field of view, spatial attention was generally directed downwards, i.e. in the area where the threat manipulation happened.

The experiment consisted of three within subject factors: *Movement* (synchronous, asynchronous), *Visibility* (visible, invisible), and *Threat* (threat, control). In the Movement condition, avatar movements were either temporo-spatially aligned with the participant’s movements (synchronous), or temporo-spatially delayed by 30 frames (asynchronous). With asynchronous movements, all body movements except head movements were delayed, and this delay was well noticeable for participants. For Visibility, we either presented a fully motion-tracked avatar (visible), or hid the avatar from the participant’s view by rendering it invisible (invisible). Threat was elicited by height exposure. A part of the floor dropped by 10 m with a speed of 5 m/s, leaving participants above the abyss on the virtual plank. An alert sound was played before the threat, followed by rumbling noise during the movement of the floor, indicating a machinery at work. We used a control stimulus and played the same sounds as with the threat stimulus, to control for confounding effects of visual and auditive attention [c.f.,^28^]. This consisted in a colour change of the area where the abyss was presented in the threat condition. The control stimulus was faded in and out, matching the temporal characteristics of the threat manipulation (videos of the different manipulations are depicted in the online material available at https://osf.io/3m4y8/).

All conditions were cross-combined, except for the asynchronous movements in the invisible condition, as it was judged to be infeasible to perform the soap-bubble-kicking task without a visual body representation when the own movements were delayed. Each participant completed six different conditions (visible-synchronous-threat, visible-synchronous-control, visible-asynchronous-threat, visible-asynchronous-control, invisible-synchronous-threat, invisible-synchronous-control). Visibility was blocked, and the block order was randomly determined. Trials within the Visibility blocks were likewise randomised.

We collected subjective measures of the embodiment components, i.e. ownership, agency, and location, by single item questions (see supplementary materials A). For this purpose, we adapted the items with the highest factor loadings of a previously used questionnaire^29^. In the invisible condition, we changed the wording from virtual body to invisible body. However, these items were only given to participants if they indicated that they had perceived an invisible body using a separate item (rating > 0). In addition to the skin conductance measure, we collected subjective fear ratings with a questionnaire item. The item on presence targeted the sense of “being there” in an environment, and was adapted from a another study^22^. For all six items (five on embodiment and presence, and one on the perception of an invisible body) we used a seven-point Likert scale ranging from 0 (“not at all”) to 6 (“very much”).

There are different measures to assess suggestibility^18,30–32^. We decided against the Sussex-Waterloo Scale of Hypnotizability (SWASH;^32^) because we did not want participant’s reactions to be biased by their associations with hypnosis^33^. The phenomenological control scale (PCS;^31^), which is an adaptation of the SWASH without framing the exercises as hypnosis, was not officially published when preparing our experiment. We therefore used the *Sensory Suggestibility Scale* (SSS;^30^) which relies on comparable exercises containing sensory suggestions without the hypnotic context. This scale consists of 10 exercises, including sham items. An example for an exercise is the suggestion of having a sweet taste in the mouth, and for a sham exercise hearing rumbling noises when covering one’s ears with the hands, i.e. a sensation which is truly perceived. We made adjustments during the translation of the scale to German. Some of those are based on the version used by Marotta et al.^18^, others were made to ensure a better fit for testing the SSS online (see supplementary materials B for a description of all scenarios used in the German adaptation). Some exercises were difficult to perform in an online version at home because of the required materials (e.g., a standardised weight, which had to be held in the hand). We therefore selected a set of 7 exercises (2 sham), referring to items 1, 2, 3, 4, 5, 8, 10^30^. Participants rated their sensations on a rating scale ranging from 0 (“no sensation”) to 4 (“very strong sensation”), whereby the labels were adjusted to fit the context of the exercise (e.g., “very strong sensation of a sweet taste”). To use the SSS in an online version, we prepared small video clips which explained and demonstrated the exercises, allowing participants to perform the exercises alongside. The order of the different exercises was randomised.

#### Procedure

Participants conducted an online survey after arriving at the laboratory. This survey was created to collect demographic data and conduct the SSS. Stereoscopic vision was tested with the Stereo Fly Test (Stereo Optical Co., Inc., Chicago, USA). Before starting the experiment, participants read the instruction for the VR experiment. We used a cover story which disguised the experimental purpose to measure the precision of VR motion tracking devices. Then, an experimenter equipped participants with electrodes for the skin conductance measure and fixed the Vive trackers to the participant’s body. After the participant mounted the HMD, we calibrated the avatar to fit the size of the participant’s physical body. We also adjusted the position of the virtual room so that the real and virtual plank coincided. The participant then performed the soap-bubble-kicking task. They started with either the visible (4 trials: synchronous-threat, synchronous-control, asynchronous-threat, asynchronous-control) or invisible (2 trials: synchronous-threat, synchronous-control) block. The threat or the control stimulus appeared after participants successfully caught 30 soap bubbles. The questions on embodiment (ownership, agency, location), presence, and fear were answered after each of the six experimental conditions. After finishing the VR experiment, participants did a post-experiment inquiry, which they again completed on their own. This inquiry included the Simulator Sickness Questionnaire (SSQ^34^) and questions about technical issues (e.g., tracking issues). The experiment had a total duration of about 90 min.

### Experiment 2: Online

#### Participants

We used a mailing list to reach out to students of Justus Liebig University Giessen to participate in the online experiment. Participants could receive course credits or opt in for a lottery of a voucher. Only participants at least 18 years old and without known neuropsychological disorders were allowed to participate in the experiment. The final sample consisted of 111 participants (95 females, 15 males, and 1 diverse), with a mean age of 22 years (SD = 4, ranging from 18 to 59). We did not have pilot data to determine the required sample size by a power analysis. Because online data can be noisy and often have a high dropout rate, we decided to collect as many participants as possible in a one-month period. We informed participants that the online survey was in line with the Declaration of Helsinki (2004) and provided a button to give consent.

#### Study design

As in the VR experiment, participants in the online experiment first finished a survey on demographic data and then proceeded to the SSS. Experimental conditions were the same as in the VR experiment (see. ‘Experiment 1: virtual reality.’). The only difference was that participants did not perform the task in VR. We explained to the participants in the online experiment that they would receive the instructions given to participants in the VR experiment. To further illustrate the VR experiment, we showed videos depicting the different conditions (see online material). After each video, participants answered the same questionnaire like in the VR experiment (see supplementary materials A). We asked participants to rate the questionnaire items as if they had participated in the VR experiment. Due to technical requirements, we did not block the Visibility condition like in the VR experiment, but randomised the presentation of all six conditions. At the end of the experiment, participants filled out a slightly adapted version of the post experiment inquiry used in the VR experiment (for example, tracking issues and the SSQ were only relevant in the VR experiment). Participants took about 40 min to complete the experiment.

### Pre-processing and data analysis

#### Exclusion VR experiment

We excluded two participants from the sample, one because of not following the instructions and the other because of having participated in the pilot experiment. Due to technical difficulties (e.g., tracking issues), we excluded values from individual blocks for the different questionnaire items (4 participants with a total of 30 excluded values).

We introduced catch items during the SSS that were meant to detect participants which did not follow the instructions. These items required to summarise the instructions or to state important materials needed for the experiment. All participants passed these items. Catch items for each segment of the SSS were used to detect participants which did not follow the experiment attentively. Participants with a wrong answer on these items were excluded from the respective item(s), but left in the sample otherwise (2 participants with a total of 2 excluded values). Two questions during the SSS required participants to feel their pulse. Participants who stated that they were not able to do so were excluded from the respective items (5 participants with a total of 5 excluded values).

#### Exclusion online experiment

To detect participants in the SSS who did not read the instruction or did not follow attentively, we used a similar procedure as in the VR experiment. Two participants could not explain the instructions of the SSS and were removed from the analysis. Three participants answered the catch items used in each segment of the SSS wrongly and were excluded from the respective items (4 excluded values). In addition, participants who did not feel their pulse were excluded from the respective items (19 participants with a total of 29 excluded values).

Similar to the SSS, we used catch items to detect participants who did not read the instructions of the VR experiment. Five participants were not able to explain these instructions and were therefore removed from the analysis. We further introduced catch items to detect trials in which participants did not follow attentively by asking questions about the presented experimental manipulations. This resulted in 63 participants being excluded from respective items (658 excluded values, i.e. 17%).

#### Pre-processing and analysis

Pre-processing and data analyses were similar for the online and the VR experiment. Pre-processing and descriptive statistics were done in Python 3.8.5, statistical data analysis in RStudio 2021.09.0 with R 4.1.0. SSS scores were calculated relative to the total number of non-excluded items. Sham items were not used for the analysis. We used a linear mixed model (LMM) with the *lmer* function from the *lmerTest* package to analyse the data for each dependent variable separately. The model included a random intercept for each participant to account for the repeated measures design, and a fixed slope.

We used a difference coding for the categorical variables with coefficient − 0.5 for online, asynchronous, invisible, control and coefficient 0.5 for VR, synchronous, visible, threat. Slopes therefore reflect the mean difference between the two factor levels. Positive slopes indicate higher ratings, and thus more pronounced effects, for the levels VR, synchronous, visible, and threat compared to their respective counter parts.

Model selection was done with the *step* function from the *lmerTest* package, which removes non-significant (*p* ≤ 0.05) model components from a fully specified model (supplementary materials C). Study, Movement, Visibility, Threat, and their respective two-way interactions specified the full model (note that it is not possible to test for an interaction between Movement and Visibility). We used Maximum Likelihood (ML) to fit the models, which (as opposed to Restricted Maximum Likelihood, REML) seems to be beneficial when interested in the fixed effects^35^, p. 29.

We assessed the model resulting from this selection process for outliers and influential cases, i.e. cases with studentised residuals outside the range of ± 3, or a cook’s distance > 1. Only the criterium for the residuals was met for the questionnaire items (number of excluded values: ownership: 3; location: 4; agency: 6; presence: 4; fear: 5). To follow up interaction effects, we calculated individual models for the online and VR data set. We then compared the model slopes for the different predictors using a *Z*-test from the function *lm_slopes_compare* from the *EMAtools* package. This allowed us to compare the strength of the effect of our manipulations between the two experiments. For the suggestibility analysis we updated those follow-up models to include additional predictors.

#### Skin conductance

We used the skin conductance measure to assure that our height exposure manipulation lead to an increased experience of threat (see supplementary materials D). Due to technical issues, skin conductance was not recorded from one participant. We used a 0.05 Hz high-pass filter together with a threshold of 0.02 µS to preprocess the data. We considered skin conductance amplitudes within a 5.5 s window, starting 0.5 s after the event onset (alert sound preceding the threat or control stimulus by 0.5 s), as event-related skin conductance response. The amplitude of the skin conductance response was log-transformed: ${\mathrm{log}}_{e}(SCR Amplitude+1)$. One value was excluded for having residuals outside the range of ± 3. The LMM fitted to the data showed that the threat compared to the control stimulus lead to a higher log-amplitude of the skin conductance response (95% CI in brackets after the slope, *b* = 0.236 [0.145–0.328], SE = 0.046, *t*(126.068) = 5.092, *p* < 0.001). This shows that the height exposure used in this experiment elicited a fear response in participants.

## Results

The goal of this study was to investigate the impact of demand characteristics on embodiment and presence. We therefore constructed two versions of the same experiment, an online and a VR version. The first is intended to assess participants’ expected experimental outcomes while passively observing a participant in the VR experiment, whereas the second directly measures the outcome while participants actively performed the VR experiment. We examined whether ratings in the online experiment matched those in the VR experiment. This was done by testing if the manipulations similarly influenced participants ratings in the online and VR experiments, which was indicated by model slopes of the same sign. If manipulations had a similar effect in both experiments, this would suggest that participants were aware of the research hypotheses. In addition, we wanted to know whether participants prone to suggestibility were more likely to report sensations of embodiment and presence. In this section, we will describe the results of the comparison between the online and VR experiments (‘Demand characteristics: Similar effects in online and VR experiments’), and then present the results on the influence of suggestibility (‘Suggestibility: Effects on ownership and location ratings’).

### Demand characteristics: similar effects in online and VR experiments

**Figure 2:**
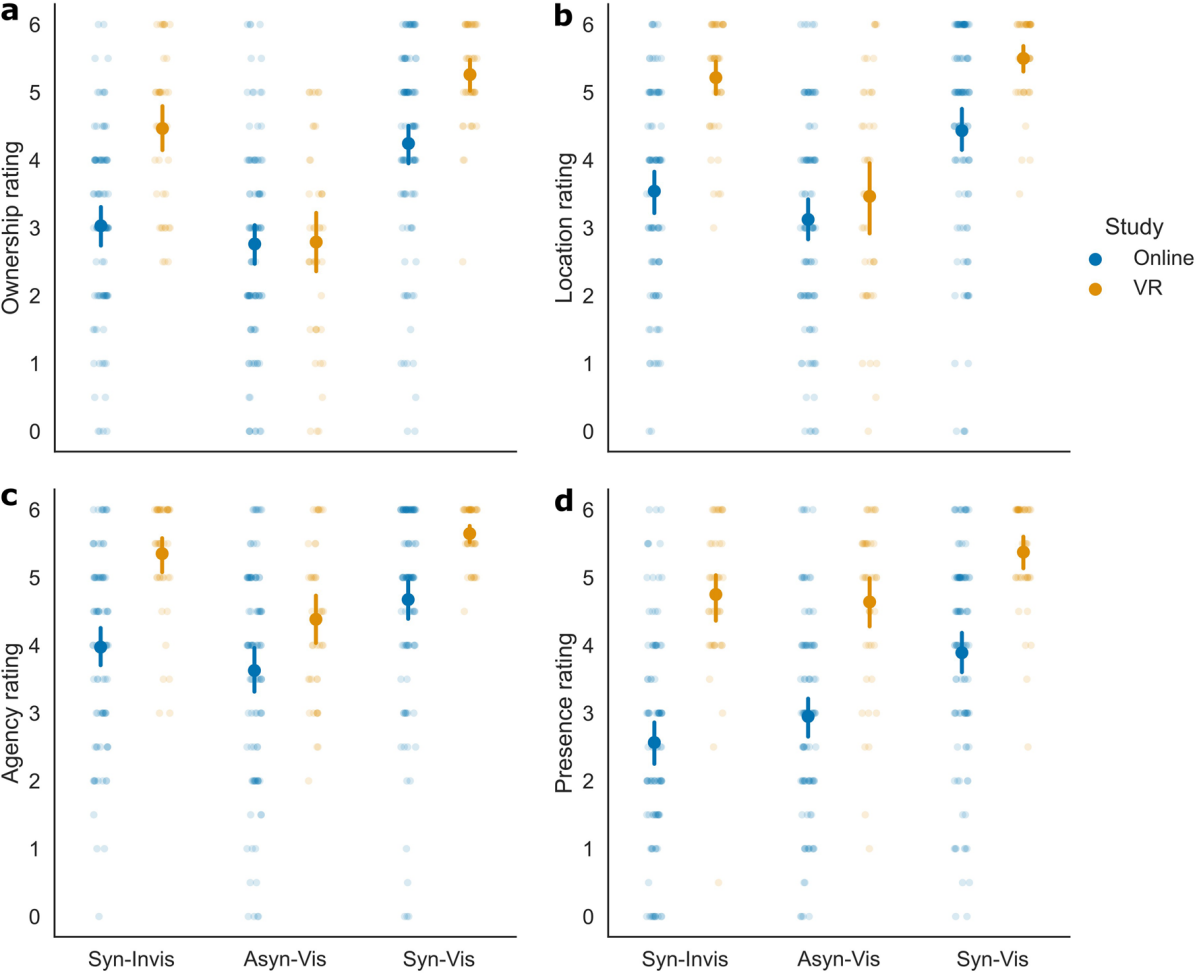
Ratings per condition for the items on (**a**) ownership, (**b**) location, (**c**) agency, and (**d**) presence. Data points are averaged over Threat per participant. Point-plots show mean values. Error bars represent the 95% within-subject CI. Individual dots represent averaged data per participant.

**Figure 3:**
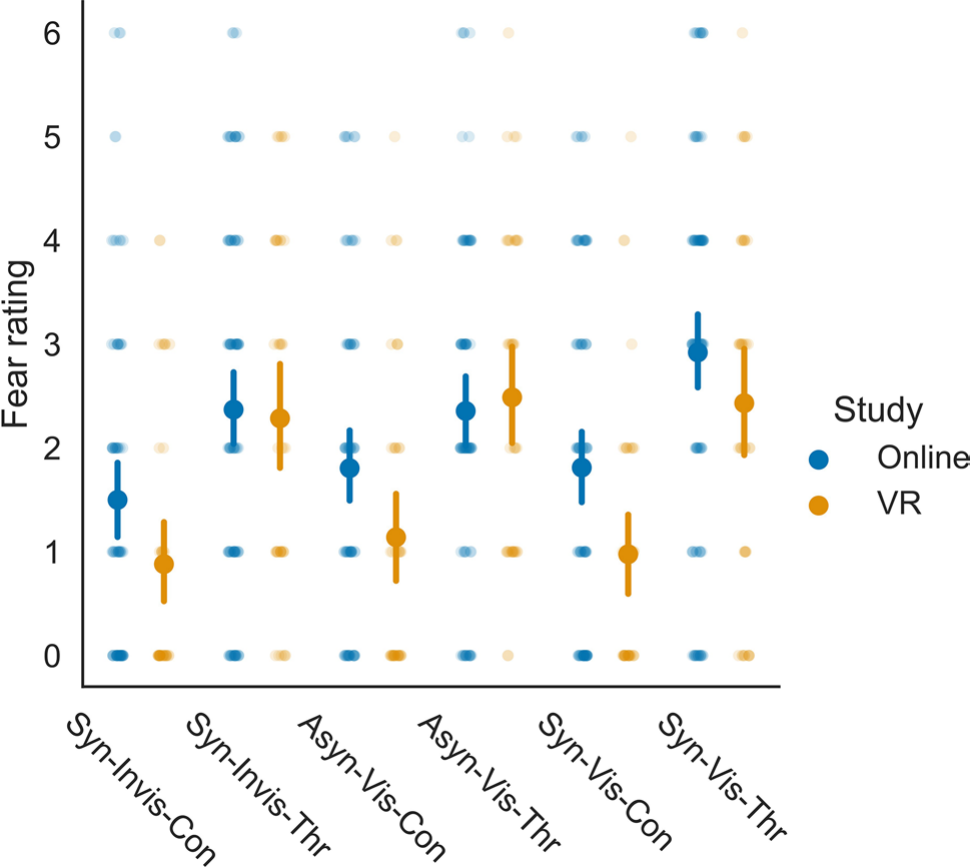
Ratings per condition for the fear item. Point-plots show mean values. Error bars represent the 95% within-subject CI. Individual dots represent ratings per participant.

Ratings differed between online and VR experiments, with generally higher ratings in the VR experiment for embodiment and presence (Fig. 2). The results further showed an effect of Movement (Asyn-Vis compared to Syn-Vis) in both experiments, and an effect of Visibility (Syn-Invis compared to Syn-Vis) more pronounced in the online experiment. Fear ratings in response to threat were similar between the online and the VR experiment (Fig. 3), however, participants rated the control stimulus as less fearful in the VR, compared to the online experiment. In the following, we will analyse the model slopes representing the differences between the two factor levels, which can be interpreted as the effect the manipulation had on the rating responses.

The slope for Study was significant for ownership, location, agency, and presence, but not for fear (Table 1). In addition, ratings were significantly influenced by Movement and Visibility for all items, and Threat only for the fear item. These effects should not be interpreted without their interactions. We found significant interactions between Study and Movement for the ownership and location items. Additionally, the interaction between Study and Visibility was significant for all items, except for the fear item, where no such interaction was included in the model. For the fear item, there was a significant interaction between Study and Threat.

**Table 1:** Fixed effects from the LMM. Statistics are reported for each item with slope (*b*), standard error (SE), degrees of freedom (df), *t*-value (*t*), *p*-value (*p*), and the 95% confidence interval (CI_*lower*_ and CI_*upper*_).

Item	Predictor	*b*	SE	df	*t*	*p*	CI_*lower*_	CI_*upper*_
Ownership	Intercept	3.233	0.106	165.844	30.636	< .001	3.025	3.441
	Study	0.788	0.211	165.844	3.735	< .001	0.373	1.205
	Movement	2.025	0.101	620.557	19.990	< .001	1.826	2.224
	Visibility	1.052	0.101	620.461	10.456	< .001	0.854	1.250
	Study:Movement	0.898	0.203	620.557	4.434	< .001	0.501	1.296
	Study:Visibility	− 0.573	0.201	620.461	− 2.845	= .005	− 0.968	− 0.177
Location	Intercept	3.798	0.111	164.439	34.122	< .001	3.579	4.017
	Study	1.091	0.223	164.439	4.900	< .001	0.652	1.530
	Movement	1.695	0.100	618.904	16.932	< .001	1.499	1.892
	Visibility	0.680	0.100	618.953	6.801	< .001	0.483	0.876
	Study:Movement	0.628	0.200	618.904	3.135	= .002	0.235	1.021
	Study:Visibility	− 0.761	0.200	618.953	− 3.806	< .001	− 1.153	− 0.368
Agency	Intercept	4.313	0.107	157.914	40.251	< .001	4.101	4.524
	Study	1.206	0.210	146.530	5.733	< .001	0.791	1.621
	Movement	1.118	0.078	618.799	14.362	< .001	0.965	1.271
	Visibility	0.569	0.081	616.536	7.026	< .001	0.410	0.728
	Study:Visibility	− 0.679	0.145	617.725	− 4.693	< .001	− 0.963	-0.395
Presence	Intercept	3.711	0.113	163.060	32.842	< .001	3.488	3.934
	Study	1.872	0.222	152.093	8.430	< .001	1.434	2.310
	Movement	0.910	0.080	644.293	11.392	< .001	0.753	1.066
	Visibility	1.005	0.082	640.809	12.309	< .001	0.845	1.166
	Study:Visibility	− 0.606	0.145	641.592	− 4.176	< .001	− 0.891	− 0.321
Fear	Intercept	1.809	0.127	161.091	14.194	< .001	1.557	2.060
	Study	− 0.431	0.249	147.769	− 1.726	= .086	− 0.923	0.061
	Movement	0.215	0.089	641.591	2.417	= .016	0.040	0.389
	Visibility	0.343	0.087	639.878	3.962	< .001	0.173	0.513
	Threat	1.176	0.077	642.450	15.208	< .001	1.024	1.328
	Study:Threat	0.441	0.155	642.448	2.851	= .004	0.137	0.745

**Figure 4:**
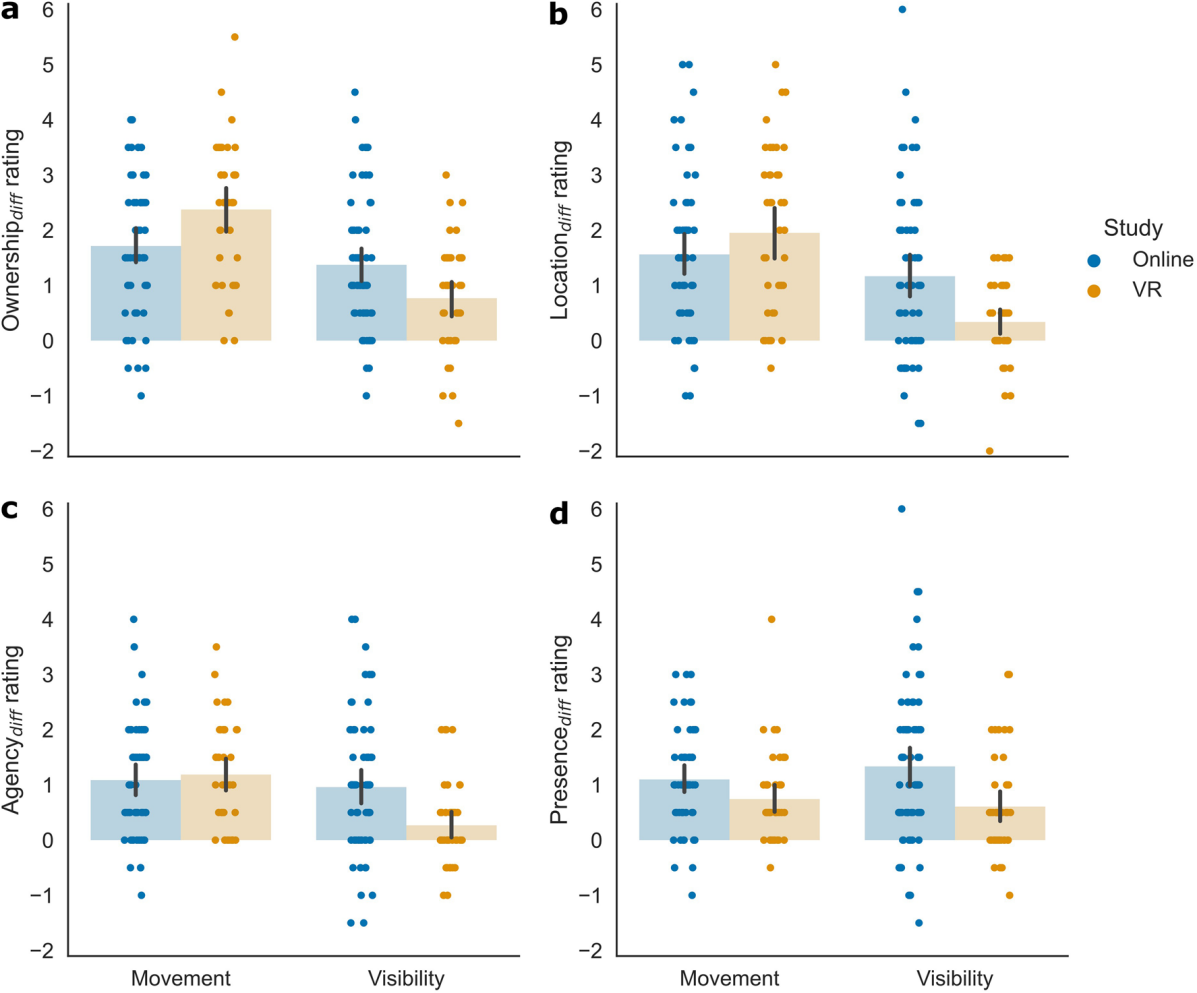
Averaged rating differences for the Movement and Visibility manipulations for the items on (**a**) ownership, (**b**) location, (**c**) agency, and (**d**) presence. Data points are averaged over Threat per participant. Values represent differences between factor levels of Movement (synchronous, asynchronous) and Visibility (visible, invisible). Positive values indicate higher ratings in synchronous and visible conditions. Bar-plots show mean values and do not represent exact model slopes. Error bars represent the 95% within-subject CI. Individual dots represent difference values per participant.

**Figure 5:**
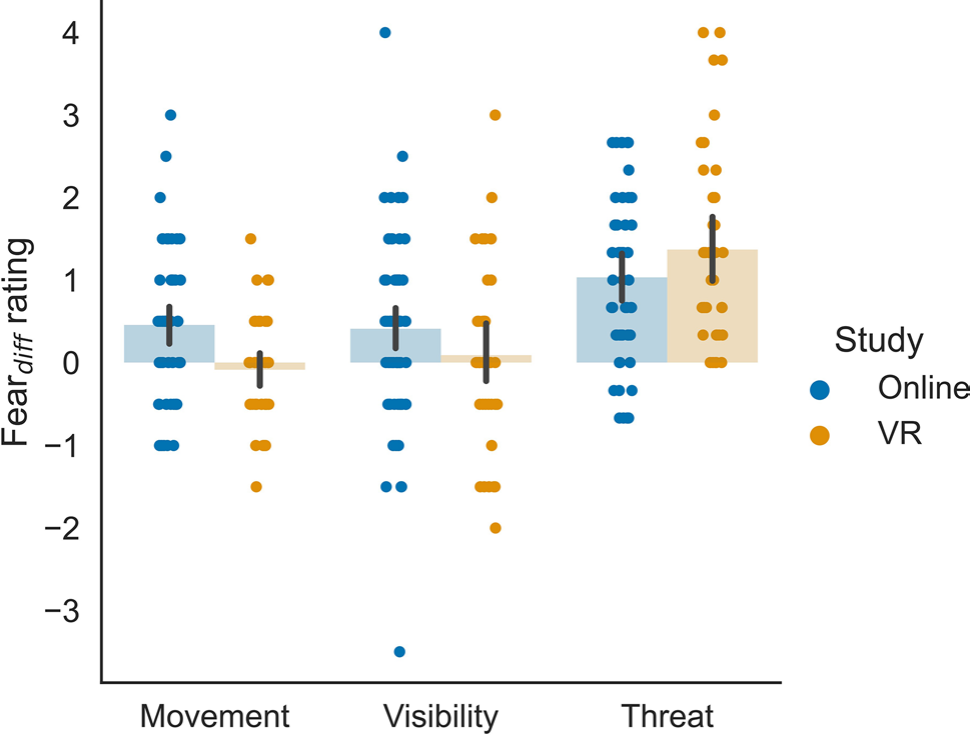
Averaged difference between Movement, Visibility and Threat manipulations for the fear item. Data points are averaged over Threat per participant for Movement and Visibility. For Threat, data points are averaged over the Visibility and Movement manipulations. Values represent differences between factor levels of Movement (synchronous, asynchronous), Visibility (visible, invisible), and Threat (threat, control). Positive values indicate higher ratings in synchronous, visible, and threat conditions. Bar-plots show mean values and do not represent exact model slopes. Error bars represent the 95% within-subject CI. Individual dots represent difference values per participant.

Follow-up models were constructed to better understand the interaction effects. Per item, we constructed an LMM for Study, including Movement and Visibility as predictors for all items, and in addition, Threat for the fear item. All model components were significant, except in the VR group, where Movement (fear item) and Visibility (location and fear item) were not significant (Table 2). Positive slopes in both the online and VR experiments suggest that manipulations influencing participants’ ratings in the online experiment also influenced their ratings in the VR experiment. Indeed, all significant slopes were positive, indicating that participants rated the items as higher when the avatar was visible (compared to invisible), the movements were synchronous (compared to asynchronous), or a threat (compared to control) stimulus appeared (see Figs. 4 and 5). This shows that participants’ expectations when rating the online material are in line with participants’ responses in the actual VR experiment, suggesting that participants were aware of the research hypotheses.

**Table 2:** Fixed effects from follow-up models for Study. Statistics are reported for each item and Study condition with slope (*b*), standard error (SE), degrees of freedom (df), *t*-value (*t*), *p*-value (*p*), and the 95% confidence interval (CI_*lower*_ and CI_*upper*_).

Item	Study	Predictor	*b*	SE	df	*t*	*p*	CI_*lower*_	CI_*upper*_
Ownership	Online	Intercept	2.835	0.126	124.159	22.581	< .001	2.587	3.083
		Movement	1.577	0.113	412.411	13.944	< .001	1.355	1.799
		Visibility	1.349	0.113	413.496	11.910	< .001	1.126	1.571
	VR	Intercept	3.629	0.138	58.412	26.259	< .001	3.353	3.904
		Movement	2.471	0.174	214.460	14.166	< .001	2.127	2.814
		Visibility	0.765	0.173	213.812	4.431	< .001	0.425	1.105
Location	Online	Intercept	3.249	0.136	123.580	23.888	< .001	2.980	3.518
		Movement	1.383	0.117	412.353	11.855	< .001	1.154	1.612
		Visibility	1.069	0.117	413.022	9.137	< .001	0.838	1.298
	VR	Intercept	4.348	0.122	60.175	35.577	< .001	4.104	4.591
		Movement	2.005	0.160	213.270	12.559	< .001	1.691	2.320
		Visibility	0.292	0.159	212.943	1.836	= .068	− 0.021	0.605
Agency	Online	Intercept	3.724	0.133	119.277	27.922	< .001	3.460	3.987
		Movement	1.060	0.098	409.110	10.792	< .001	0.867	1.252
		Visibility	0.887	0.099	410.118	8.964	< .001	0.692	1.081
	VR	Intercept	4.887	0.098	59.881	50.008	< .001	4.692	5.081
		Movement	1.236	0.126	213.725	9.840	< .001	0.989	1.483
		Visibility	0.283	0.124	213.092	2.274	= .024	0.038	0.527
Presence	Online	Intercept	2.753	0.133	122.235	20.635	< .001	2.490	3.017
		Movement	0.991	0.105	430.823	9.456	< .001	0.785	1.197
		Visibility	1.345	0.101	429.103	13.319	< .001	1.147	1.544
	VR	Intercept	4.689	0.143	49.422	32.830	< .001	4.403	4.974
		Movement	0.748	0.115	214.616	6.489	< .001	0.521	0.974
		Visibility	0.623	0.114	214.339	5.463	< .001	0.398	0.847
Fear	Online	Intercept	1.979	0.146	119.136	13.567	< .001	1.691	2.267
		Movement	0.366	0.109	431.518	3.375	= .001	0.153	0.579
		Visibility	0.437	0.105	430.059	4.185	< .001	0.232	0.643
		Threat	0.959	0.090	438.077	10.659	< .001	0.782	1.135
	VR	Intercept	1.681	0.184	49.783	9.117	< .001	1.312	2.049
		Movement	− 0.092	0.152	210.771	− 0.606	= .545	− 0.391	0.207
		Visibility	0.137	0.152	210.510	0.905	= .366	− 0.161	0.436
		Threat	1.402	0.124	210.397	11.297	< .001	1.157	1.646

The follow-up models showed that all significant slopes were positive, which indicates that differences in the magnitude of the effects, as reflected in the steepness of the slopes, drive the significant interactions involving Study in the overall models. To assess whether the manipulations led to a higher increase in the ratings in the online or VR experiment, we compared the intercepts and slopes between the respective LMMs with a *Z*-test. Differences in slopes indicate that our manipulations had a more pronounced effect in either the online or VR experiment. The intercepts differed significantly between online and VR experiments for all comparisons, except for the fear item (ownership: *Z* = 4.252, *p* < 0.001; location: *Z* = 6.008, *p* < 0.001; agency: *Z* = 7.034, *p* < 0.001; presence: *Z* = 9.903, *p* < 0.001; fear: *Z* = − 1.268, *p* = 0.205). For Movement, the increase in ownership and location ratings was significantly higher in the VR experiment (ownership: *Z* = 4.299, *p* < 0.001; location: *Z* = 3.145, *p* = 0.002). This suggests that Movement had a stronger effect on ownership and location ratings in the VR than the online experiment. For Visibility, ratings for the different items (ownership, location, agency, and presence) showed a significantly stronger increase in the online experiment (ownership: *Z* = − 2.828, *p* = 0.005; location: *Z* = − 3.932, *p* < 0.001; agency: *Z* = − 3.804, *p* < 0.001; presence: *Z* = − 4.744, *p* < 0.001). This suggests that Visibility increased the ratings in the online experiment more than in the VR experiment. For the subjective location ratings, we already observed that Visibility only influenced ratings in the online experiment, but not in the VR experiment (see Table 2). Additionally, Threat showed a significantly stronger increase in the fear rating in the VR experiment (*Z* = 2.892, *p* = 0.004). This effect is likely based on differences between ratings in the control condition of the online and VR experiments (see Fig. 3), with lower subjective fear ratings in the VR group, when a control stimulus was presented. Despite this, the fear ratings in the online and VR experiments were highly similar. For Movement, the increase in the fear ratings was higher in the online experiment (*Z* = − 2.454, *p* = 0.014), reflecting that Movement had an effect in the online, but not in the VR experiment (see Table 2). However, as no interaction between Study and Movement was present in the overall model (see Table 1), this result should be interpreted with caution. All other comparisons of slopes between the online and the VR experiment (Movement for the agency and the presence item; Visibility for the fear item) were not significant (*p* ≥ 0.103), as could be expected from the missing interactions in the overall model. Altogether, we observed differences between slopes of the online and VR experiments. Movement had a stronger effect on embodiment (except agency) ratings in the VR experiment, whereas Visibility had a stronger effect on embodiment and presence ratings in the online experiment. Most importantly, the slopes in both experiments had the same sign, indicating that the manipulations had a similar effect on participants’ ratings in the online and VR experiments. This suggests that demand characteristics affected our results on embodiment and presence.

### Suggestibility: Effects on ownership and location ratings

Our results from the online experiment show that participants can accurately rate which manipulations influence embodiment and presence, and might therefore know the underlying hypotheses. Participants prone to suggestibility might more strongly change their experiences in accordance to the perceived demand characteristics of the experiment [c.f.,^8,9^]. To uncover the role of suggestibility, we reanalysed the models for each experiment and included Suggestibility as an additional predictor. Two-way interactions between Suggestibility and the other model components were considered as predictors as well. For the VR experiment, some predictors had no influence on the rating (Visibility for the location and fear item, Movement for the fear item, see Table 2), and were therefore removed from the model before adding Suggestibility as a new predictor. An ANOVA was used to compare models with different predictors.

**Figure 6:**
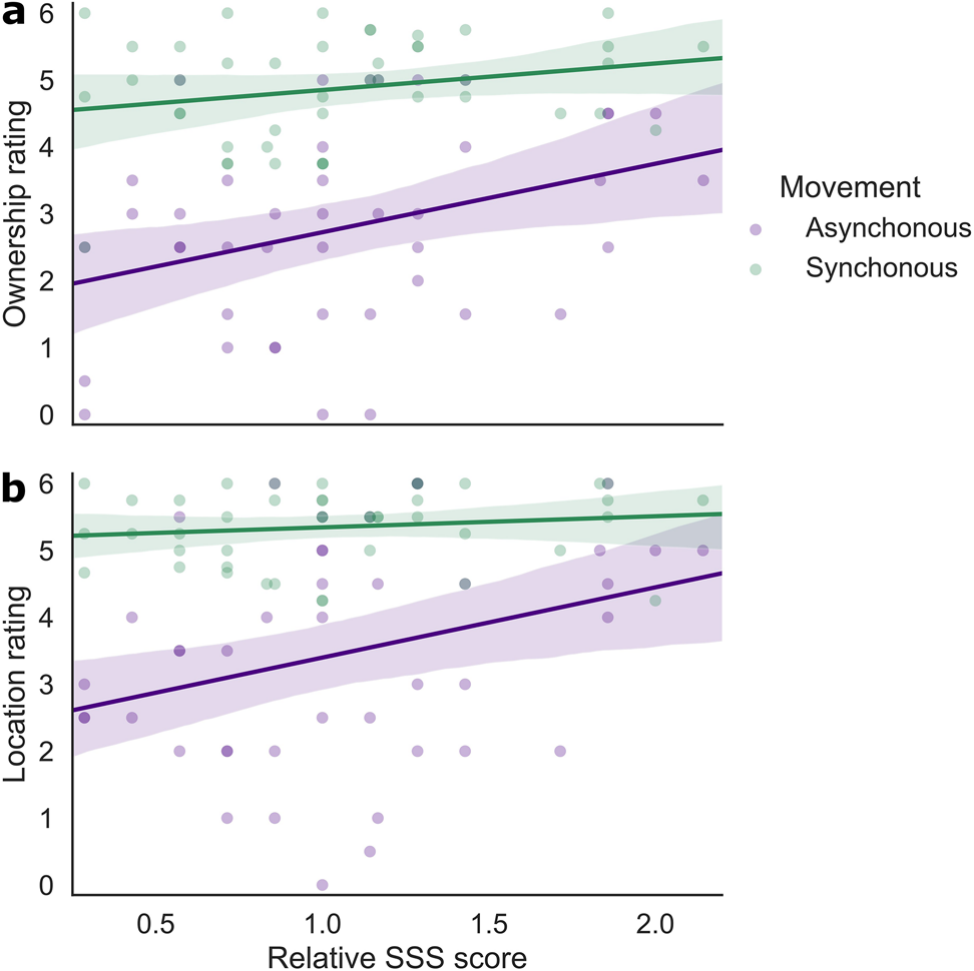
Suggestibility and Movement in the VR experiment for items on (**a**) ownership and (**b**) location. For each participant, data points are averaged over Visibility and Threat, per Movement condition. The regression plot is based on the data and does not represent the exact model slopes. Shaded areas represent the 95% within-subject CI. Individual dots represent averaged data per participant.

Suggestibility (ownership: *b* = 0.736 [0.220, 1.253], SE = 0.258, *t*(47.773) = 2.848, *p* = 0.006; location: *b* = 0.619 [0.160, 1.080], *t*(47.893) = 2.692, *p* = 0.010) as well as the interaction between Suggestibility and Movement (ownership: *b* = − 0.625 [− 1.237, − 0.013], SE = 0.311, *t*(214.305) = − 2.011, *p* = 0.046; location: *b* = − 0.929 [− 1.487, − 0.370], SE = 0.284, *t*(213.411) = − 3.271, *p* = 0.001) were significant predictors for the ownership and the location ratings in the VR experiment. All other included predictors remained significant (*p* < 0.001, see supplementary materials E). A follow-up model on the interaction showed that ratings on ownership and location increased with participants’ suggestibility in the asynchronous movement condition (ownership: *b* = 1.025 [0.143, 1.906], SE = 0.440, *t*(42.998) = 2.329, *p* = 0.025; location: *b* = 1.057 [0.071, 2.042], SE = 0.491, *t*(42.320) = 2.151, *p* = 0.037), but not in the synchronous movement condition (ownership: *b* = 0.410 [− 0.082, 0.900], SE = 0.245, *t*(43.749) = 1.674, *p* = 0.101; location: *b* = 0.155 [− 0.189, 0.501], SE = 0.172, *t*(44.135) = 0.900, *p* = 0.373). Figure 6 illustrates the relationship between Suggestibility and the subjective ratings indicating that participants with higher scores in the SSS also gave higher ratings when answering items on ownership and location in the asynchronous movement condition.

## Discussion

We investigated the impact of demand characteristics by comparing participants’ ratings from experiments in which they rated the questionnaire items by only watching a participant performing the experiment to ratings in the actual experiment. In line with previous findings [e.g.,^8^], we showed that participants’ expectations about the experimental outcomes on embodiment and presence mirror the corresponding research hypotheses. This shows that demand characteristics do not only influence results in RHI experiments but could also affect embodiment measures in this sensorimotor VR task. This finding was not restricted to embodiment, but was also found for presence. When comparing the results of the online and VR experiments, we observed differences in the magnitude of the effects. Movement had a stronger influence on embodiment (except agency) in the VR experiment, whereas Visibility had a stronger effect on embodiment and presence in the online experiment. The effect of Threat on the fear ratings was also more pronounced in the VR experiment. In addition, suggestibility predicted subjective ratings on ownership and location in the asynchronous movement condition suggesting that the relationship between suggestibility and embodiment ratings differed depending on the Movement manipulation.

### The effect of demand characteristics

We applied different experimental manipulations to investigate to which extent embodiment and presence are affected by demand characteristics. The key finding of our study was that participants were able to judge the influence of the different manipulations on the questionnaire ratings in the online version of the experiment. This was true for all three manipulations: Movement, Visibility, and Threat. These results indicate that participants in the online experiment were aware of the research hypotheses and that participants in the VR experiment might have gained the same knowledge.

In the VR experiment, we observed an effect of Movement on embodiment, with higher ratings in the synchronous movement condition. This is in line with previous results showing that visuomotor^36^ and visuotactile^36,37^ synchrony can induce embodiment. In addition, we observed an effect of Movement on presence, supporting the finding that performing body movements in VR is positively related to presence^38^. We also found an effect of avatar Visibility on embodiment (ownership and agency), with higher ratings when the avatar was visible, supporting previous results^39^. Nevertheless, it has been shown that participants can also perceive embodiment of an invisible body^40^. An effect of Visibility was also observed for presence, with higher ratings when the avatar is visible. This is in line with findings that having a virtual body representation promotes presence [c.f.,^23,41^]. Contrary to a previous study^42^, the fear ratings in the VR experiment seemed to be only influenced by Threat and not by our other manipulations.

Importantly, the effects of Movement, Visibility, and Threat were also present in the online experiment. This finding is in line with previous studies claiming that embodiment measures might be confounded by demand characteristics, i.e. the knowledge about the research hypotheses^8,13,14^. We extended these results to presence where we observed similar effects. These results highlight the importance to consider demand characteristics when investigating embodiment and presence.

We also observed differences in the magnitude of the effects, with Movement having a generally more pronounced effect on embodiment ratings in the VR experiment, and Visibility on embodiment and presence ratings in the online experiment. We do not believe that mere context effects, i.e. rating questionnaire items online in a private atmosphere compared to a laboratory setting where an experimenter is present, strongly influenced the results. This is supported by a previous study showing that a behavioural task in a laboratory and an online experiment yielded similar results^43^. However, rating questionnaire items online or in VR might involve different perceptual and decision-making processes. The response modalities differ in so far that participants either reported their experienced sensations in the VR experiment or their expected sensations in the online experiment. Overall, these differences in magnitude stand against the clear similarities of both experiments indicating that demand characteristics cannot be ruled out when interpreting results on embodiment and presence. This does not mean that results on embodiment and presence are necessarily based on demand characteristics. Participants could recognise the experiment’s hypotheses, but still genuinely experience embodiment and presence.

The results reported here could be limited by the fact that some participants might have perceived the control stimulus as abyss, especially when presented with this stimulus first. There could therefore be a tendency to rate the control stimulus higher, if it was presented before the threat stimulus. This is unlikely in the online experiment as we used a catch item to exclude participants who could not discriminate between the control and threat stimuli. For the VR experiment, we observed similar fear ratings for presenting the control stimulus before or after the first threat stimulus, which also speaks against such a bias.

### The effect of suggestibility

Guessing the research hypotheses could affect the results, e.g. by a response bias or by participants’ suggestibility. We measured participants’ suggestibility and observed an effect on ownership and location ratings for asynchronous movements in the VR experiment. We did not find this effect in our online experiment, possibly due to different requirements of the experiment. Participants rated their experienced sensations in the VR experiment, whereas they rated their expected sensations in the online experiment. It is conceivable that suggestibility, as the ability to change ones experiences to match perceived demands^9,17^, is less likely to affect the more cognitive tasks as applied in the online experiment.

Previous findings showed an effect of suggestibility on ownership under synchronous stroking of the rubber hand^18^. Although, we used a different experimental design, we would have expected similar results, especially as we used the same measure of suggestibility. The lack of an influence of suggestibility on subjective ratings in the synchronous movement condition is likely caused by a ceiling effect. This suggests that questionnaire items in both conditions can be confounded by suggestibility, which is in line with findings reporting an effect of suggestibility for synchronous and asynchronous stimulations^9,44^.

Comparing illusion and control conditions, e.g. synchronous and asynchronous conditions, could control for suggestibility. Ehrsson et al.^20^ used this approach when reanalysing data by Lush et al.^9^ and found no relation between suggestibility and subjective ratings on the RHI. In our study, suggestibility predicted subjective ratings on ownership and location only in the asynchronous movement condition. In this case, investigating differences between both conditions cannot control for suggestibility. To identify conditions which are differently influenced by suggestibility, small exercises measuring individual suggestibility should be applied [c.f.,^18,30–32^].

Our results on suggestibility are limited by the fact that some faint noise from a server room was audible while participants performed the SSS in the VR experiment. This noise might have interfered with acoustic tasks in the SSS making it difficult to detect an influence from suggestibility on participants’ ratings. We nevertheless found such an influence for ownership and location. In addition, the SSS scores did not differ between the online and the VR experiment, making it unlikely that the noise from the server room biased our results.

We only used a subset of SSS exercises. If psychometric properties of this subset are different from the overall properties is difficult to evaluate because the study on which we based our version of the SSS did not report psychometric properties. The PCS^31^ reports psychometric properties and could be an alternative for future experiments. It was not officially published when we created this study.

As randomisation of experimental blocks was not possible in the online experiment, we always presented the SSS in the beginning. The online and the VR experiment should closely resemble each other, which is why we decided to also present the SSS in the VR experiment first. In theory, this could have led to carry over effects, which, however, would be consistent across experiments.

### Challenges in embodiment and presence research

Demand characteristics challenge research on embodiment and presence. Here, we show that participants’ ratings in the online experiment are in line with the reported sensations of participants in the actual VR experiment. This indicates that demand characteristics could have confounded the ratings on embodiment and presence. Participants’ ratings could reflect their knowledge about the experiment rather than reports of embodiment and presence sensations per se. This could express itself in a response bias, e.g. when participants actively try to produce responses confirming the hypotheses^10^, or might provide the demands against which suggestible participants can match their experiences [e.g.,^9^].

Demand characteristics are not the only factor reducing the validity of subjective measures. Questionnaire items are also the only way participants can express themselves, and sensations reported could in theory be a consequence of surveying participants^45^. To reach conclusive results on embodiment and presence, we therefore need to consider additional methodological factors concerning standardisations of experimental designs [c.f.,^46,47^], concise and measurable definitions of constructs, and the use of objective measures.

Finding suitable objective measures for embodiment and presence turns out to be a challenge. First, demand characteristics might also confound objective measures such as proprioceptive drift and skin conductance^13^. For example, if participants’ experiences change according to demand characteristics^9,12^, this might lead to a change in their physiological response^48^. It is therefore questionable how effective these measures are in preventing the confounding effect of demand characteristics. Second, results from physiological measures are not consistently reported to match self-reports of embodiment [c.f.,^49^] and presence [c.f.,^24^]. Accordingly, we found that the skin conductance response to threat was unaffected by manipulations affecting embodiment and presence ratings. This might suggest that physiological and subjective measures might rely on different processes. This view is supported by recent findings which show that objective and subjective measures on ownership and agency might partly rely on different information^50^. It is therefore difficult to indicate which measures are best suited to study embodiment and presence.

Apart from these challenges, ratings on embodiment and presence are more or less influenced by the same manipulations. This makes it difficult to separate the two constructs experimentally, and it is unclear, to which extent participants are able to differentiate their reported sensations when rating questionnaire items. These findings support current ideas suggesting that embodiment and presence could be explained within a common framework^51,52^.

### Conclusion

In this study, we directly compared embodiment and presence ratings from a VR study and its online version. We showed that participants’ ratings in the online experiment were similar to the responses in the VR experiment indicating that participants were aware of the research hypotheses. This replicates previous findings on demand characteristics for embodiment, and extends these to presence. It remains an open question how exactly participants’ knowledge influences subjective and objective measures on embodiment and presence. Future research is challenged to find methods eliminating the confounding effects of demand characteristics, e.g. by finding and applying effective control procedures.

## Supplementary Information

Supplementary Information.

## Data Availability

The videos of the different conditions as well as data created during the analysis of this study are available at: https://osf.io/3m4y8/.

## References

[1] Kilteni K, Groten R, Slater M (2012). The sense of embodiment in virtual reality. Presence Teleoperators Virtual Environ..

[2] Botvinick M, Cohen J (1998). Rubber hands 'feel' touch that eyes see. Nature.

[3] Guterstam A, Larsson DEO, Zeberg H, Ehrsson HH (2019). Multisensory correlations—not tactile expectations—determine the sense of body ownership. PLoS ONE.

[4] Tsakiris M (2010). My body in the brain: A neurocognitive model of body-ownership. Neuropsychologia.

[5] Tsakiris M, Carpenter L, James D, Fotopoulou A (2010). Hands only illusion: Multisensory integration elicits sense of ownership for body parts but not for non-corporeal objects. Exp. Brain Res..

[6] Ehrsson HH, Spence C, Passingham RE (2004). That's my hand! activity in premotor cortex reflects feeling of ownership of a limb. Science.

[7] Yizhar O (2021). Body ownership of anatomically implausible hands in virtual reality. Front. Human Neurosci..

[8] Lush P (2020). Demand characteristics confound the rubber hand illusion. Collabra Psychol..

[9] Lush P (2020). Trait phenomenological control predicts experience of mirror synaesthesia and the rubber hand illusion. Nat. Commun..

[10] Nichols AL, Maner JK (2008). The good-subject effect: Investigating participant demand characteristics. J. Gen. Psychol..

[11] Orne MT (1962). On the social psychology of the psychological experiment. With particular reference to demand characteristics and their implications. Am. Psychol..

[12] Corneille, O. & Lush, P. Sixty years after Orne’s American Psychologist article: A conceptual analysis of “Demand Characteristics”. Preprint at 10.31234/osf.io/jqyvx (2022). 10.1177/10888683221104368 35801624

[13] Lush P, Seth AK, Dienes Z (2021). Hypothesis awareness confounds asynchronous control conditions in indirect measures of the rubber hand illusion. R. Soc. Open Sci..

[14] Reader AT (2022). What do participants expect to experience in the rubber hand illusion? A conceptual replication of Lush (2020). Collabra Psychol..

[15] Young SD, Adelstein BD, Ellis SR (2007). Demand characteristics in assessing motion sickness in a virtual environment: Or does taking a motion sickness questionnaire make you sick?. IEEE Trans. Visual. Comput. Graphics.

[16] Coles, N. A., Gaertner, L., Frohlich, B., Larsen, J. T. & Basnight-Brown, D. M. (2022) Fact or artifact Demand characteristics and participants’ beliefs can moderate but do not fully account for the effects of facial feedback on emotional experience. *PsyArXiv.*10.31234/osf.io/br4y9 10.1037/pspa0000316 35617225

[17] Dienes Z (2022). Phenomenological control as cold control. Psychol. Conscious. Theory Res. Practice..

[18] Marotta A, Tinazzi M, Cavedini C, Zampini M, Fiorio M (2016). Individual differences in the rubber hand illusion are related to sensory suggestibility. PLoS ONE.

[19] Roseboom W, Lush P (2022). Serious problems with interpreting rubber hand “illusion” experiments. Collabra Psychol..

[20] Ehrsson HH, Fotopoulou A, Radziun D, Longo MR, Tsakiris M (2022). No specific relationship between hypnotic suggestibility and the rubber hand illusion. Nat. Commun..

[21] Lush P, Seth AK (2022). Reply to: No specific relationship between hypnotic suggestibility and the rubber hand illusion. Nat. Commun..

[22] Usoh M, Catena E, Arman S, Slater M (2000). Using presence questionnaires in reality. Presence Teleoperators Virtual Environ..

[23] Slater M, Spanlang B, Corominas D (2010). Simulating virtual environments within virtual environments as the basis for a psychophysics of presence. ACM Trans. Graphics.

[24] Eftekharifar S, Thaler A, Troje NF (2020). Contribution of motion parallax and stereopsis to the sense of presence in virtual reality. J. Percept. Imaging.

[25] Faul F, Erdfelder E, Lang A-G, Buchner A (2007). G*Power 3: A flexible statistical power analysis program for the social, behavioral, and biomedical sciences. Behav. Res. Methods.

[26] Slater M, Spanlang B, Sanchez-Vives MV, Blanke O (2010). First person experience of body transfer in virtual reality. PLoS ONE.

[27] Peck TC, Seinfeld S, Aglioti SM, Slater M (2013). Putting yourself in the skin of a black avatar reduces implicit racial bias. Conscious. Cognit..

[28] Remington RW, Johnston JC, Yantis S (1992). Involuntary attentional capture by abrupt onsets. Percept. Psychophys..

[29] Longo MR, Schüür F, Kammers MPM, Tsakiris M, Haggard P (2008). What is embodiment? a psychometric approach. Cognition.

[30] Lund K (2015). The magnitude of placebo analgesia effects depends on how they are conceptualized. J. Psychosom. Res..

[31] Lush P, Scott RB, Seth AK, Dienes Z (2021). The phenomenological control scale: Measuring the capacity for creating illusory nonvolition, hallucination and delusion. Collabra Psychol..

[32] Lush P, Moga G, McLatchie N, Dienes Z (2018). The sussex-waterloo scale of hypnotizability (SWASH): Measuring capacity for altering conscious experience. Neurosci. Consciou..

[33] Michael RB, Garry M, Kirsch I (2012). Suggestion, cognition, and behavior. Curr. Dir. Psychol. Sci..

[34] Kennedy RS, Lane NE, Berbaum KS, Lilienthal MG (1993). Simulator sickness questionnaire: An enhanced method for quantifying simulator sickness. Int. J. Aviat. Psychol..

[35] Twisk JWR (2010). Applied multilevel analysis. A practical guide.

[36] Kalckert A, Ehrsson HH (2014). The moving rubber hand illusion revisited: Comparing movements and visuotactile stimulation to induce illusory ownership. Conscious. Cogn..

[37] O’Kane SH, Ehrsson HH (2021). The contribution of stimulating multiple body parts simultaneously to the illusion of owning an entire artificial body. PLoS ONE.

[38] Slater M, Steed A, McCarthy J, Maringelli F (1998). The influence of body movement on subjective presence in virtual environments. Hum. Factors.

[39] Martini M, Kilteni K, Maselli A, Sanchez-Vives MV (2015). The body fades away: Investigating the effects of transparency of an embodied virtual body on pain threshold and body ownership. Sci. Rep..

[40] Guterstam A, Abdulkarim Z, Ehrsson HH (2015). Illusory ownership of an invisible body reduces autonomic and subjective social anxiety responses. Sci. Rep..

[41] Steed A, Pan Y, Watson Z, Slater M (2018). “We wait”—the impact of character responsiveness and self embodiment on presence and interest in an immersive news experience. Front. Robot. AI.

[42] Ehrsson HH, Wiech K, Weiskopf N, Dolan RJ, Passingham RE (2007). Threatening a rubber hand that you feel is yours elicits a cortical anxiety response. Proc. Natl. Acad. Sci..

[43] Casler K, Bickel L, Hackett E (2013). Separate but equal? a comparison of participants and data gathered via amazon’s MTurk, social media, and face-to-face behavioral testing. Comput. Hum. Behav..

[44] Fiorio M, Modenese M, Cesari P (2020). The rubber hand illusion in hypnosis provides new insights into the sense of body ownership. Sci. Rep..

[45] Slater M (2004). How colorful was your day? Why questionnaires cannot assess presence in virtual environments. Presence Teleoperators Virtual Environ..

[46] Sivasubramaniam AK, Ng J-H, Chan H, Yang JKY, Kalckert A (2021). The super-stroker—an open-source tool to induce the rubber hand illusion. Psychol. Conscious. Theory Res. Pract..

[47] Riemer M, Trojan J, Beauchamp M, Fuchs X (2019). The rubber hand universe: On the impact of methodological differences in the rubber hand illusion. Neurosci. Biobehav. Rev..

[48] Hägni K (2008). Observing virtual arms that you imagine are yours increases the galvanic skin response to an unexpected threat. PLoS ONE.

[49] Kokkinara E, Kilteni K, Blom KJ, Slater M (2016). First person perspective of seated participants over a walking virtual body leads to illusory agency over the walking. Sci. Rep..

[50] Ma K, Qu J, Yang L, Zhao W, Hommel B (2021). Explicit and implicit measures of body ownership and agency: Affected by the same manipulations and yet independent. Exp. Brain Res..

[51] Nostadt N, Abbink DA, Christ O, Beckerle P (2020). Embodiment, presence, and their intersections: Teleoperation and beyond. ACM Trans. Hum. Rob. Interact..

[52] Forster P-P, Karimpur H, Fiehler K (2022). Why we should rethink our approach to embodiment and presence. Front. Virtual Real..

