# Supplementary material for: Demand characteristics challenge effects in embodiment and presence
Source: Sci Rep. 2022 Aug 18;12:14084. doi: 10.1038/s41598-022-18160-5 (PMC9387424; doi:10.1038/s41598-022-18160-5)
Supplement: Supplementary file 1 — Supplementary Information. [file 41598_2022_18160_MOESM1_ESM.pdf]

Supplementary materials:

Demand characteristics challenge effects in embodiment and presence

Pierre-Pascal Forster[1,2], Harun Karimpur[1,2], Katja Fiehler[1,2]

[1]Experimental Psychology, Justus Liebig University, Giessen, Germany

[2]Center for Mind Brain and Behavior, University Marburg and Justus Liebig University Giessen, Germany

A: Questionnaire items

	German adaptation	Translation (of German adaptation)	English original	Reference
**Ownership**	Während des Experiments schien es, als beginne der virtuelle Körper meinem echten Körper zu entsprechen.	During the experiment, it seemed like the virtual body began to resembled my real body.	...it seemed like the rubber hand began to resemble my real hand.	Longo et al., 2008, p. 987
**Location**	Während des Experiments schien es, als befände sich der virtuelle Körper an der Position meines Körpers.	During the experiment, it seemed like the virtual body was in the location where my body was.	...it seemed like the rubber hand was in the location where my hand was.	Longo et al., 2008, p. 987
**Agency**	Während des Experiments schien es, als könnte ich den virtuellen Körper bewegen.	During the experiment, it seemed like I could have moved the virtual body.	...it seemed like I could have moved the rubber hand if I had wanted.	Longo et al., 2008, p. 987
**Presence**	Ich hatte das Gefühl „dort zu sein“ im virtuellen Labor.	I had a sense of “being there” in the virtual laboratory.	[…] I had a sense of “being there” in the office space.	Usoh et al., 2000, p. 499
**Fear**	Als sich der Boden verändert hat, habe ich Furcht empfunden.	When the bottom had changed, I experienced fear.		No previous use
**Invisible body**	Während des Experiments schien es, als hätte ich einen unsichtbaren Körper.	During the experiment, it seemed like I had an invisible body.		No previous use

Table S1. Questionnaire items.

B: Scenarios of the Sensory Suggestibility Scale (SSS)

These are descriptions of the SSS scenarios used in the present study. The German adaptation of the SSS is based on Lund et al. (2015) with some of the changes made in accordance to Marotta et al. (2016).

1. Ocean Sounds (sham)

Participants were instructed to listen to a sound similar to enclosing one's ears with a shell. We then asked them to cover their ears with both hands and to listen if they could hear a rushing noise.

2. Provocation of a salty and a sweet taste

We explained participants that different tastes are experienced at different parts of one's tongue. Salty tastes would be localised at the tongue's periphery, whereas sweet tastes at the tip of the tongue. We further told them that pressure to the tongue would suffice to elicit those sensations. Participants were then asked to first press the periphery and then the tip of their tongue against their teeth, and to report any sensations of a salty or sweet taste.

3. Tingling in the hand (sham)

We explained that shaking the hand violently can lead to a tingling sensation due to the increased blood circulation. Participants then shook their hand and reported if they felt a tingling sensation.

4. Perception of one's pulse

We told participants that one sometimes strongly feels one's pulse, e.g. when doing sports. To get an idea of their pulse, participants measured their pulse at their temple. After removing their fingers from their temple, we asked them to report if they could still feel their pulse on their fingertips.

5. Acoustic perception of one's pulse

Hearing a tactile stimulus was introduced as an aftereffect of synaesthesia. Participants again felt their pulse at their temple. They then closed their ear with their finger to shield them from outside noises and listened if they could hear their pulse.

8. Perception of the finger contours and a bluish colour

This exercise consisted of two parts. The first part was meant to produce an aftereffect of one's finger contours. We instructed participants to observe the contours of their fingers and then to concentrate on if they could still see those contours after closing their eyes.

For the second part we told participants that a soft pressure onto one's closed eyes can elicit a bluish colour sensation. Participants then softly pressed their fingers against their closed eyes and rated their sensation of the colour blue.

10. Perception of warmth on the face

We instructed participants that shining a light into one's face leads to the sensation of warmth. To ensure that they used an LED having a low warmth discharge, we told them to use the torch light of their phone. Participants then shone with the torch light into their face and rated the sensation of warmth.

C: Model selection

Interaction effects are indicated by a colon, e.g., Movement:Study specifies the interaction between Movement and Study.

**Predictor**	**Eliminated**	**Sum of Sq.**	**Mean Sq.**	**df*num***	**df*den***	***F***	***p***
Visibility:Threat	1	0.028	0.028	1	622.838	0.023	=.879
Movement:Threat	2	0.082	0.082	1	624.364	0.067	=.796
Study:Threat	3	0.937	0.937	1	629.170	0.767	=.382
Threat	4	0.026	0.026	1	639.102	0.021	=.884
Study:Movement	0	21.311	21.311	1	623.489	17.412	<.001
Study:Visibility	0	11.099	11.099	1	623.029	9.068	=.003

Table S2. Model selection for the ownership item.

**Predictor**	**Eliminated**	**Sum of Sq.**	**Mean Sq.**	**df*num***	**df*den***	***F***	***p***
Study:Threat	1	0.155	0.155	1	628.931	0.127	=.722
Visibility:Threat	2	0.426	0.426	1	622.940	0.348	=.555
Movement:Threat	3	1.551	1.551	1	624.228	1.267	=.261
Threat	4	0.608	0.608	1	637.963	0.496	=.482
Study:Movement	0	11.325	11.325	1	623.557	9.222	=.002
Study:Visibility	0	12.489	12.489	1	623.170	10.170	=.001

Table S3. Model selection location item.

**Predictor**	**Eliminated**	**Sum of Sq.**	**Mean Sq.**	**df*num***	**df*den***	***F***	***p***
Visibility:Threat	1	0.053	0.053	1	620.501	0.061	=.805
Study:Threat	2	0.087	0.087	1	625.308	0.100	=.752
Movement:Threat	3	0.831	0.831	1	621.421	0.955	=.329
Study:Movement	4	1.301	1.301	1	620.931	1.493	=.222
Threat	5	1.687	1.687	1	632.444	1.931	=.165
Movement	0	166.237	166.237	1	623.968	189.785	<.001
Study:Visibility	0	21.201	21.201	1	622.679	24.205	<.001

Table S4. Model selection agency item.

**Predictor**	**Eliminated**	**Sum of Sq.**	**Mean Sq.**	**df***num*	**df***den*	***F***	***p***
Visibility:Threat	1	0.149	0.149	1	644.524	0.172	=.679
Study:Threat	2	0.218	0.218	1	649.329	0.252	=.616
Movement:Threat	3	0.882	0.882	1	646.087	1.017	=.314
Threat	4	0.350	0.350	1	655.651	0.403	=.526
Study:Movement	5	1.730	1.730	1	645.858	1.991	=.159
Movement	0	106.344	106.344	1	648.457	121.967	<.001
Study:Visibility	0	12.968	12.968	1	645.572	14.873	<.001

Table S5. Model selection presence item.

**Predictor**	**Eliminated**	**Sum of Sq.**	**Mean Sq.**	**df***num*	**df***den*	***F***	***p***
Visibility:Threat	1	0.924	0.924	1	642.826	0.862	=.354
Movement:Threat	2	2.785	2.785	1	644.439	2.596	=.108
Study:Visibility	3	3.275	3.275	1	642.598	3.041	=.082
Study:Movement	4	2.774	2.774	1	645.428	2.563	=.110
Movement	0	7.364	7.364	1	646.704	6.774	=.009
Visibility	0	17.050	17.050	1	644.640	15.685	<.001
Study:Threat	0	6.163	6.163	1	647.593	5.670	=.018

Table S6. Model selection fear item.

D: Skin conductance response

Predictor	Eliminated	Sum of Sq.	Mean Sq.	df_{num}	df_{den}	F	p
Visibility:Threat	1	0.003	0.003	1	130.399	0.041	=.841
Threat:Suggestibility	2	0.027	0.027	1	126.963	0.323	=.571
Movement:Threat	3	0.034	0.034	1	128.881	0.408	=.524
Suggestibility:SSQ	4	0.043	0.043	1	43.955	0.522	=.474
Threat:SSQ	5	0.045	0.045	1	125.274	0.543	=.462
Movement:SSQ	6	0.037	0.037	1	126.183	0.440	=.508
Visibility:Suggestibility	7	0.063	0.063	1	128.792	0.750	=.388
Movement:Suggestibility	8	0.061	0.061	1	126.369	0.724	=.396
Movement	9	0.017	0.017	1	127.492	0.206	=.651
Suggestibility	10	0.086	0.086	1	42.871	1.022	=.318
Threat	0	2.419	2.419	1	126.953	28.633	<.001
Visibility:SSQ	0	0.366	0.366	1	126.237	4.326	=.040

Table S7. Model selection log-amplitude skin conductance. The score from the simulator questionnaire (SSQ, Kennedy et al., 1993) was included to control for a potential confound of simulator sickness on skin conductance response. The effect of Visibility and the Interaction between Visibility and the SSQ score was found to be non-significant in follow-up models and consequently removed from the overall model.

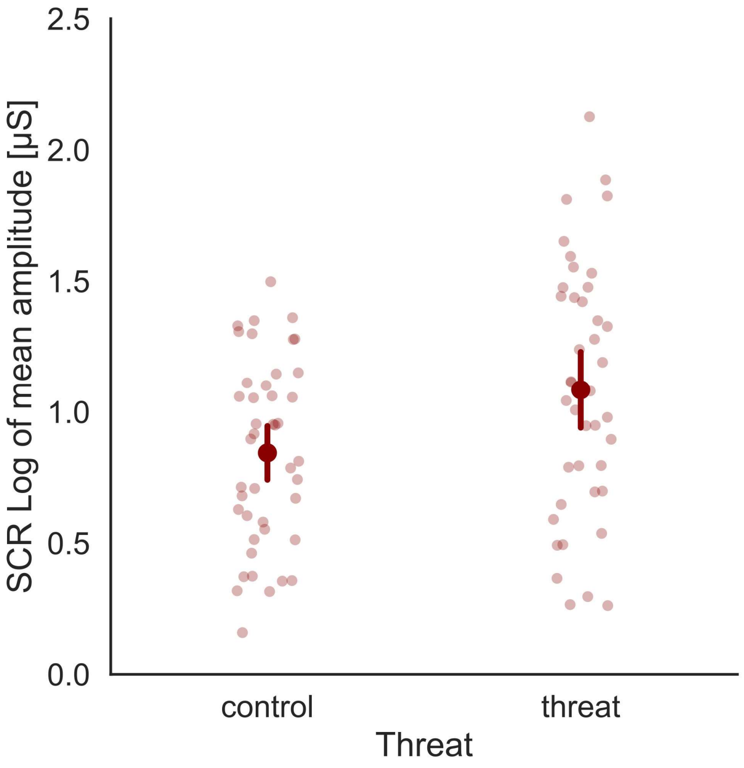

Figure S1. Skin conductance response for Threat. For the figure, data points are first averaged across conditions per participants and then log-transformed. Point plots show mean values and individual dots represent values per participant. Error bars represent the 95% within-subject CI.

E: Suggestibility analysis

Item	Predictor	*b*	SE	df	*t*	*p*	CI_{lower}	CI_{upper}
Ownership	Intercept	2.851	0.303	50.081	9.417	<.001	2.245	3.455
	Movement	3.133	0.372	215.118	8.413	<.001	2.400	3.866
	Visibility	0.765	0.171	213.992	4.476	<.001	0.428	1.101
	Suggestibility	0.736	0.258	47.773	2.848	=.006	0.220	1.253
	Movement:Suggestibility	-0.625	0.311	214.305	-2.011	=.046	-1.237	-0.013
Location	Intercept	3.767	0.267	48.346	14.108	<.001	3.232	4.301
	Movement	2.847	0.331	214.331	8.598	<.001	2.195	3.499
	Suggestibility	0.619	0.230	47.893	2.692	=.010	0.160	1.080
	Movement:Suggestibility	-0.929	0.284	213.411	-3.271	=.001	-1.487	-0.370

Table S8. Fixed effects from the suggestibility models in the VR experiment. Statistics are reported for each item with slope (*b*), standard error (SE), degrees of freedom (df), *t*-value (*t*), *p*-value (*p*), and the 95% confidence interval (CI_{lower} and CI_{upper}).

References

Kennedy, R. S., Lane, N. E., Berbaum, K. S., & Lilienthal, M. G. (1993). Simulator sickness questionnaire: An enhanced method for quantifying simulator sickness. *The International Journal of Aviation Psychology*, *3*(3), 203–220. https://doi.org/10.1207/s15327108ijap0303_3

Longo, M. R., Schüür, F., Kammers, M. P. M., Tsakiris, M., & Haggard, P. (2008). What is embodiment? A psychometric approach. *Cognition*, *107*(3), 978–998. https://doi.org/10.1016/j.cognition.2007.12.004

Lund, K., Petersen, G. L., Erlandsen, M., Pascalis, V. D., Vase, L., Jensen, T. S., & Finnerup, N. B. (2015). The magnitude of placebo analgesia effects depends on how they are conceptualized. *Journal of Psychosomatic Research*, *79*(6), 663–668. https://doi.org/10.1016/j.jpsychores.2015.05.002

Marotta, A., Tinazzi, M., Cavedini, C., Zampini, M., & Fiorio, M. (2016). Individual differences in the rubber hand illusion are related to sensory suggestibility. *PLoS ONE*, *11*(12), Article e0168489, 1-12. https://doi.org/10.1371/journal.pone.0168489

Usoh, M., Catena, E., Arman, S., & Slater, M. (2000). Using presence questionnaires in reality. *Presence: Teleoperators and Virtual Environments*, *9*(5), 497–503. https://doi.org/10.1162/105474600566989
